# Evaluation of remote teaching in master programs in the Oral Pathology and Oral Medicine during the COVID-19 pandemic. Brazilian multicentric study

**DOI:** 10.4317/medoral.26004

**Published:** 2023-07-10

**Authors:** Fabrício Emanuel Soares de Oliveira, Nelson Pereira Marques, Daniella Reis Barbosa Martelli, Lucas Alves Trindade França, Pablo Agustin Vargas, Ludmila Ketlen Soares de Oliveira, Maria Isabela Soares de Alencar Monteiro, Maria Cássia Ferreira de Aguiar, Jean Nunes dos Santos, Janete Dias Almeida, Hercilio Martelli Júnior

**Affiliations:** 1 Postgraduate Program in Primary Health Care. State University of Montes Claros (UNIMONTES), Montes Claros, Minas Gerais, Brazil; 2 Department of Oral Diagnosis, Piracicaba Dental School, Universidade Estadual de Campinas (FOP-UNICAMP), Piracicaba, São Paulo, Brazil; 3 Oral Pathology and Oral Medicine, Dental School, State University of Montes Claros (Unimontes), Montes Claros, Minas Gerais, Brazil; 4 Dental School. FIPMOC University Center, Montes Claros, Minas Gerais, Brazil; 5 Department of Oral Pathology and Surgery, School of Dentistry, Federal University of Minas Gerais (UFMG), Belo Horizonte, Brazil; 6 Laboratory of Oral Surgical Pathology, School of Dentistry, Universidade Federal da Bahia (UFBA), Salvador, Bahia, Brazil; 7 Department of Bioscience and Oral Diagnosis, Institute of Science and Technology of São José dos Campos, São Paulo State University (UNESP), São José dos Campos, São Paulo, Brazil

## Abstract

**Background:**

With the COVID-19 pandemic, there was a need to adopt online teaching methods in a generalized and sudden way, a situation that led to unprecedented changes in the routine of post-graduate students and research development. This study aimed to analyze the evaluation of remote teaching by graduates of master's degrees and advisors in master's programs in the Oral Pathology and Oral Medicine who needed to adapt to a remote teaching methodology in the pandemic.

**Material and Methods:**

This quantitative study evaluated the remote teaching in the perception of master's graduates and advisors from postgraduate programs in Oral Pathology and Oral Medicine. Data were collected through an online Google forms® questionnaire.

**Results:**

Participated in the study 14 master graduates and 14 master's advisors. Master graduates evaluated that the professors had a good performance (*p*=0.001), that the duration of the classes was adequate (*p*=0.015), that the interaction with professors was satisfactory (*p*=0.007), that they had good interaction with the advisor (*p*=0.001), that they were satisfied with the remote guidance process (*p*=0.038) and that face-to-face practical activities were missed (*p*=0.002). Master's advisors reported satisfaction with remote teaching, good adaptation (*p*=0.018) and motivation for remote teaching (*p*=0.016), they evaluated that students were cooperative in activities (*p*=0.019) and that face-to-face practical activities were missed (*p*=0.002).

**Conclusions:**

Despite the difficulties, remote teaching proved to be an effective alternative to face-to-face teaching.

** Key words:**COVID-19, pandemics, education, graduate, education, dental. education, distance.

## Introduction

The COVID-19 pandemic has affected education systems worldwide at all levels of education. Many countries had to interrupt school activities, closing educational institutions indefinitely, leaving students with their school activities paralyzed (1).

The suspension of face-to-face activities in educational institutions and their replacement by emergency remote teaching negatively affected students from all over the world at different income levels, however low-income families suffered the greatest impact due to lack of access to the means of communication, accentuating social inequalities (2).

At higher and postgraduate levels, access to remote education is greater when compared to other education degrees. It is worth noting, however, that in addition to socioeconomic issues, autonomy in studies, the ability to use digital resources by teachers and academics, the ability to manage time, difficulty in finding a job, delays in licensing and certification, changes at work, anxiety and depression at work are factors that may have affected graduates of undergraduate and graduate courses during the COVID-19 pandemic (3,4).

At the beginning of the COVID-19 pandemic in Brazil, it was observed that university students underwent unprecedented changes in their lifestyle, which can impair academic performance, such as low productivity, changes in mood, changes in sleep routine, and increased consumption of alcohol and medication (5). In this sense, it is necessary for universities to adopt measures to prevent pedagogical damage to guarantee the quality of teaching, even with the changes imposed by the pandemic (4).

The pandemic had a significant impact on dental education worldwide. Hands-on activities are crucial in teaching dentistry, as they allow students to apply theoretical knowledge in real-life situations and acquire technical dental skills, which have been affected by the COVID-19 pandemic, depending on observing, assisting, training, and performing under supervision in dental laboratories (6). However, a mixed teaching approach can be effective in dental education, as demonstrated by a study (7).

In view of the above, this is the first study in the literature to analyze the evaluation of remote teaching by graduates of master's degrees and advisors in master's programs in Oral Pathology (OP) and Oral Medicine (OM) who needed to adapt to a remote teaching methodology due to the COVID-19 pandemic.

## Material and Methods

This study evaluated the remote teaching in the perception of master's graduates and advisors in master's courses who adopted remote teaching to adapt to the social distancing measures imposed by the COVID-19 pandemic. The research had as inclusion criteria to be a graduate with master's degrees that started the course in the first semester of 2020 and professors who guided master's students in the same period from postgraduate programs in OP and OM. This research was approved by the Institutional Ethics Committee (#57703622.4.0000.5146).

Data were collected through an online Google forms® questionnaire. Two data collection instruments were developed, one for master's graduates and the other for mentoring professors. The questionnaires are similar, with adaptations for each audience, organized into five blocks of questions: sociodemographic issues, satisfaction with remote teaching, teacher-student interaction, anxiety about remote teaching, and research development. The questions in the block’s satisfaction with online teaching and teacher-student interaction were adapted from the questionnaire used in Li *et al*. (8). research, which evaluated remote teaching of medicine and nursing courses in China during the COVID-19 pandemic.

The blocks satisfaction with remote teaching, teacher-student interaction, and anxiety about remote teaching had response options on a Likert scale with the following items scored from 1 to 5: strongly disagree [1]; disagree [2]; neutral, neither agree nor disagree [3], agree [4], strongly agree [5]. The questions in the teacher-student interaction block had yes or no answer options.

Data collection took place between August and November 2022 through contact with the advisors of the post-graduate programs in OP and OM from Universidade Federal da Bahia (UFBA) located in the state of Bahia, Universidade Federal de Minas Gerais (UFMG) located in the state of Minas Gerais, Universidade Estadual Paulista (UNESP) and Universidade Estadual de Campinas (FOP-UNICAMP) both located in the state of São Paulo and so that the invitation to participate in the survey and the link to the questionnaire could be sent to possible participants in the survey, thus being a convenience sampling.

The data obtained from the survey were entered into the program Statistical Package for the Social Sciences for Windows, Inc.®, USA (SPSS). Descriptive data analysis was performed with absolute and relative frequencies for categorical variables and mean and standard deviation for the age variable. The normality of the sample was assessed using the Kolmogorov-Smirnov test, and the Wilcoxon test was applied to test the null hypotheses (H0 - remote teaching had no effect on graduates and master's advisors, having the response option “neutral, neither agree nor disagree” as a reference for the expected median) and alternative (H1 - remote teaching affected graduates and master's advisors), adopting the significance level of *p* <0.005.

## Results

The study included 14 graduates and 14 master's advisors with a majority of women in both groups, 64.3% and 57.1%, respectively. The average age was 27.9 for graduates and 50.2 for advisors. The four graduate programs are from public institutions in three Brazilian states (Bahia, Minas Gerais and São Paulo) (Table 1).

Regarding the evaluation of remote teaching by master's graduates, there was statistical significance in the items: not being familiar with remote teaching tools before the master's degree (*p*=0.003), liking studying independently (*p*=0.046) and participating in classes (*p*=0.032) in the block of remote learning satisfaction; in the student-teacher interaction block, they evaluated that the professors had a good performance (*p*=0.001), that the duration of the classes was adequate (*p*=0.015), that the interaction with professors was satisfactory (*p*=0.007), that the professors were available for extra class communications (*p*<0.001), that they had facility with remote teaching tools (*p*=0.002), that they had good interaction with the advisor (*p*=0.001), that they were satisfied with the remote guidance process (*p*=0.038) and that face-to-face practical activities were missed (*p*=0.002); in the anxiety block regarding remote learning, they evaluated that they were confident (*p*=0.002) and that they were not anxious when studying using computers or other electronic devices (*p*=0.019), that they do not avoid using electronic devices (*p*=0.002) and that they were not anxious when using internet (*p*=0.003) (Table 2).

**Table 1:**
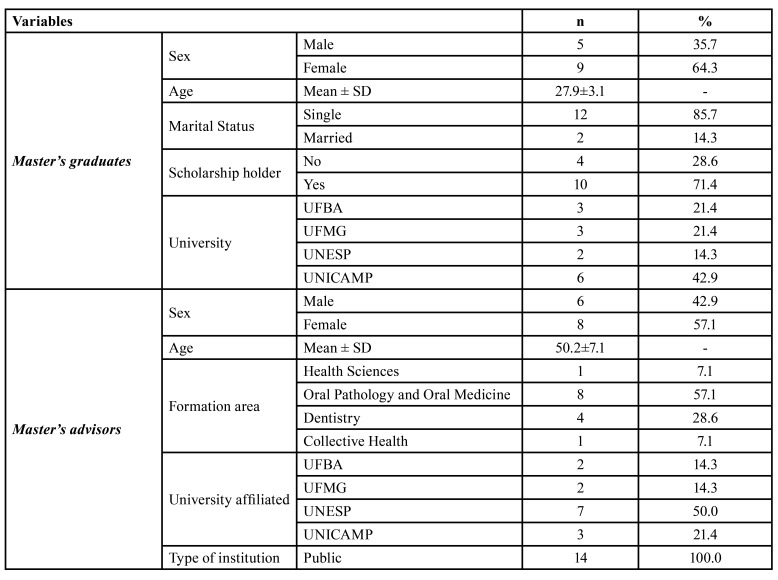
Sociodemographic characteristics of master's graduates (*n*=14) and master's advisors (*n*=14).

**Table 2:**
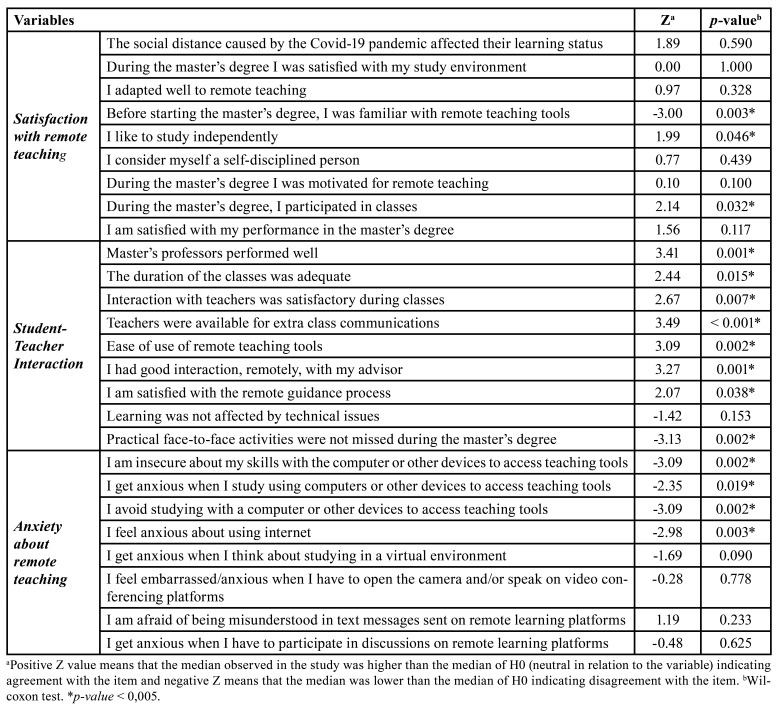
Evaluation of remote teaching in the period of the COVID-19 pandemic by master's graduates (*n*=14).

Master's advisors evaluated the following items with statistical significance: in the block satisfaction with remote teaching, good adaptation (*p*=0.018), and motivation for remote teaching (*p*=0.016); in the student-teacher interaction block, they evaluated that students were cooperative in activities (*p*=0.019) and that face-to-face practical activity was missed (*p*= 0.002); in the anxiety block regarding remote learning, they evaluated that they were not anxious when studying using computers (*p*=0.001) and did not avoid using them (*p*=0.001), that they did not feel anxious when using internet (*p*=0.002) or when they think about studying in a virtual environment (*p*=0.001), that they did not feel embarrassed when opening the camera on remote teaching platforms (*p*=0.001), that they were not afraid of being misunderstood by text messages (*p*=0.038) and that they did not feel anxious when participating in discussions in a virtual environment (*p*=0.001) (Table 3).

Regarding research development, 28.6% of graduates and advisors reported the need to interrupt the research and start another project due to the COVID-19 pandemic, 57.1% of graduates and 78.6% of advisors had to change their research projects due to the pandemic and 50.0% of both groups had to change the project research schedule. Most graduates (85.7%) reported that the pandemic affected their studies and research, in addition to having delayed research development and master's thesis defense in 42.9% of cases (Table 4).

**Table 3:**
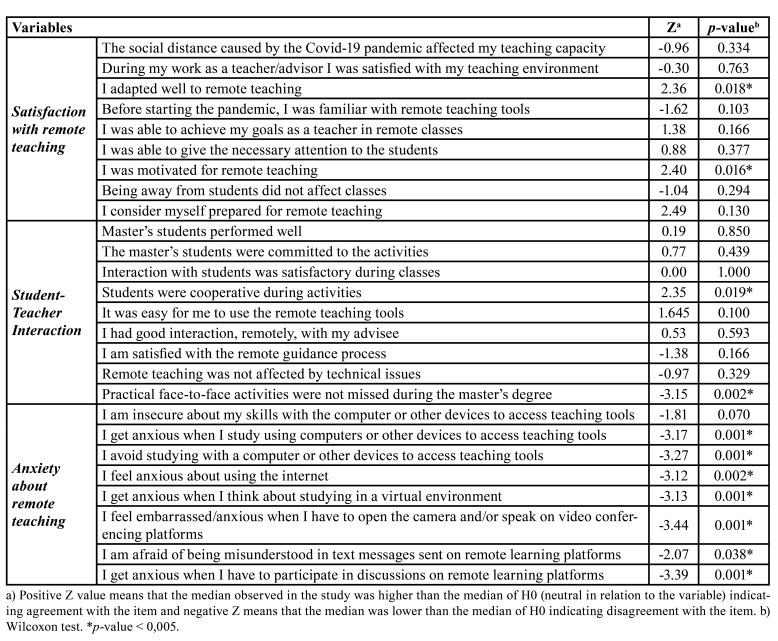
Evaluation of remote teaching in the period of the COVID-19 pandemic by master's advisors (*n*=14).

**Table 4:**
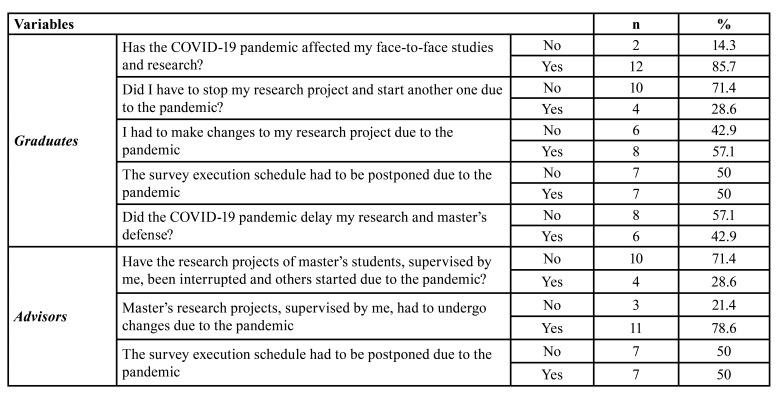
Characteristics related to the development of research by master's graduates and advisors (*n*=14).

## Discussion

In this study, aspects related to the socio-demographic profile of master's degree graduates and advisors, satisfaction with remote teaching, teacher-student interaction, anxiety about remote teaching, and changes in research development during the COVID-19 pandemic of four Brazilian public universities were analyzed. Notably, the increase in graduate and postgraduate distance education courses, including Stricto sensu, has become a reality in Brazil and the world in recent years. With the COVID-19 pandemic, there was a need to adopt this mode of teaching in a generalized and sudden way (9,10), being observed a scarcity of studies on the subject analyzed in this work.

It is important to highlight the changes adopted by the universities participating in this research. At UFBA, in early 2020, face-to-face classes were suspended, and online classes began in the second half of 2020 with the support of a virtual learning platform. Only surgical pathology activities were carried out in person, with alternate groups, as decided by the institution's committee. In 2022, activities restarted in person (Personal Communication, Santos, JN).

UFMG suspended face-to-face classes in March 2020, adopting the online format in the second half of the same year. Practical classes resumed in the second half of 2021, with restrictions on the number of students and hybrid mode of operation. All defenses moved online and remain in the same format until now. During 2022, the hybrid modality predominated, and in 2023, full face-to-face clinical activities were resumed (Personal Communication, Aguiar, MCF).

At UNESP, during the pandemic, all credits, as well as postgraduate defenses, carried out online through Google Meet platform®. Patient care was maintained through tele-triage (https://rare-rice-8265.glideapp.io/), tele-guidance, teleconsultation, and teleinterconsultation. In addition to the virtual learning environment and Moodle/Google Meet tools, a Virtual Microscopy Laboratory (https://ead.ict.unesp.br/course/view.php?id=279) stands out, which is a database containing digitized slides, available on the Moodle platform, where it is possible to view the slide information and use a virtual microscope to zoom in at different levels, making it similar to a real microscope. Clinical consultations and laboratory routines in Oral Pathology, with the participation of in-person graduate students, were resumed flexibly under the guidelines of the UNESP Covid-19 Committee (Personal Communication, Almeida, JD).

FOP-UNICAMP had already been using Google Classroom since 2016. During the pandemic, Google Meet® was adopted for online postgraduate courses. All credits and thesis defenses migrated to this format. In addition, adaptations were made to laboratory routines, and remote histopathology was introduced, allowing the continuity of academic training (11).

Regarding satisfaction with remote teaching, the graduates reported that they liked to study independently and were participative in classes. In contrast, the advisors reported good adaptation and motivation for remote teaching. In fact, remote teaching has advantages such as low cost, flexible schedules and convenience which can provide greater ease of adaptation to teaching (12). Satisfaction with remote teaching, an important factor in evaluating the effectiveness of a teaching program, demonstrates a variation in the results found in scientific literature, with studies that have obtained high satisfaction (13-15) and others that found low satisfaction (8,16,17). Study conducted during the pandemic found a positive perception of remote teaching by students and teachers in medicine courses (18), and a qualitative study demonstrates the advantages of remote teaching, supporting its use in dental and medical area (19).

In Brazil, postgraduate programs are mostly in-person, however, in other countries remote teaching modality has already been adopted at this level of education. For example, a study conducted at a university in the United States of America, carried out in 2016 (20) evaluated a master's program in dental hygiene education, with favorable results for remote teaching. An investigation of European dental students' perspectives on the impact of COVID-19 demonstrated their concern for clinical experience and skills (21).

The lack of familiarity with remote teaching tools was reported by master's graduates in this study, showing that they had to adapt to the technologies. During the COVID-19 pandemic, various technologies were used to meet the demand for emergency remote learning, including applications, websites, videoconferencing platforms and virtual reality tools, and many people do not have a deep understanding of these tools (13,18,22). However, no major difficulties were observed in adapting to remote teaching tools.

Regarding student-teacher interaction, graduates rated positively in most items with statistical significance, and advisors reported good cooperation from students in activities. However, both groups reported the lack of practical activities as a negative aspect. Good interaction between teachers and students is crucial for a successful teaching-learning process, and satisfaction with the teaching, both for teachers and students, is strongly related to mutual influence between the two. Factors such as discipline, cooperation, participation, availability, attention, patience, empathy, motivation and ease of communication favor the teaching-learning process (8).

The absence of practical activities was found to be a similar result to this investigation in a study that evaluated remote teaching in medicine courses during the COVID-19 pandemic, in which students also reported dissatisfaction with the inability to perform practical activities because skills necessary for professional practice can best be taught in clinics and laboratories (23). Nevertheless, the study conducted by Silveira *et al*., 2022 (24), discusses the importance of laboratory and clinical activities in the teaching of Pathology and Oral Medicine for Brazilian dental students. Regarding anxiety related to remote teaching, both graduates and advisors evaluated that the use of digital tools and virtual interaction did not cause discomfort or anxiety, showing security in using electronic devices and using teaching platforms. Despite this result, studies have found evidence of a negative impact on students’ mental health during the pandemic. For example, in the study by Corrêa *et al*. (25), 61.7% of postgraduate students reported anxiety symptoms. Other studies (26-29) found a higher prevalence of symptoms of mental disorders in higher education students and in health professionals than in the general population.

More than half of the projects developed by the graduates and advisors had to undergo changes to adapt to the measures imposed by the pandemic. Nearly a third, in both groups, had their projects made unfeasible by the pandemic and had to start another. In addition, half of the participants had to change the research implementation schedule. A similar result was found in a review that describe the negative impact of the pandemic in scientific research, causing limitations mainly in clinical studies (30).

Some limitations can be observed in this investigation, such as remote data collection that can introduce a potential selection bias; the sample is restricted to a few postgraduate programs from some Brazilian universities, which leads to a lack of representativeness of the studied population; and convenience sampling that prevents generalizations about the studied population.

In general, this study showed that the evaluation of master's graduates and advisors was positive in most items evaluated. However, difficulties were also reported, showing that the emergency remote teaching adopted during the COVID-19 pandemic, in addition to overcoming the difficulty imposed by social distancing for teaching, has the potential to be an effective teaching method in graduate programs.
